# Effect of sex in the MRMT-1 model of cancer-induced bone pain

**DOI:** 10.12688/f1000research.6827.3

**Published:** 2015-11-16

**Authors:** Sarah Falk, Tamara Al-Dihaissy, Laura Mezzanotte, Anne-Marie Heegaard

**Affiliations:** 1 Department of Drug Design and Pharmacology, University of Copenhagen, Copenhagen, 2100, Denmark; 2 Department of Radiology, Leiden University Medical Center, Leiden, 2333 ZA, Netherlands

**Keywords:** Cancer-induced bone pain, sex differences, in vivo model, x-ray, pain behavior, bioluminescence

## Abstract

An overwhelming amount of evidence demonstrates sex-induced variation in pain processing, and has thus increased the focus on sex as an essential parameter for optimization of in vivo models in pain research. Mammary cancer cells are often used to model metastatic bone pain in vivo, and are commonly used in both males and females. Here we demonstrate that compared to male rats, female rats have an increased capacity for recovery following inoculation of MRMT-1 mammary cells, thus potentially causing a sex-dependent bias in interpretation of the data.

## Introduction

A crucial step in translational research is development of animal models that can accurately mimic the human condition in relation to both symptoms and underlying mechanisms ^1, 2^. To understand the molecular mechanism of complex human disease, and thereby create disease-specific treatment, the models need to be reproducible and with a high predictive value, requiring a detailed knowledge of the expected variation in the models used. Cancer-induced bone pain is a highly complex pain state involving cancer cells, bone cells, immune cells as well as neuronal and non-neuronal processing in the periphery and at spinal and supraspinal levels ^3^. Numerous studies have reported on variation in the more than 45 animal models that have been used to model the pain state ^4^. The most common variations are related to cell lines, species or injection site used ^4^, and in addition we have previously demonstrated that sex does also affect the model ^5^, thereby emphasizing the need to consider sex bias.

The majority of preclinical pain research is still performed using male animals, despite an overwhelming amount of both human and animal data demonstrating significant sex variations ^6– 8^. Conversely, within the field of bone cancer pain, preclinical researchers have had more focus on the issue, likely due to a more intuitive choice of sex, based on the origin of the cancer cells used. A meta-analysis of studies on cancer-induced bone pain demonstrated that 49% of the studies used males, 22% used females and 29% did not report the sex of the animals used ^4^. These numbers are indeed more balanced than the numbers previously reported, revealing that 79% of all animal studies published in PAIN from 1996 to 2005 were using male animals, whereas only 8% used females ^8^. However, in this study we demonstrate that not only is sex an integral factor to be considered when choosing an animal model, intrinsic sex-dependent variation has to be carefully analyzed in order to avoid bias. The data presented in this study suggest, that in the MRMT-1-Luc2 carcinoma cell model of metastatic bone cancer pain, a sex-dependent bias related to recovery is skewing the behavioral responses observed in females to a greater extent compared to males, potentially masking effects in studies where female animals are used to model the pain state.

## Methods

Experiments were performed on 10 male and 10 female Sprague-Dawley rats (Taconic M&B, Denmark) group-housed in cages under a 12-h alternating light/dark cycle with ad libitum access to food and water. In addition, unpublished data from four previous independent in-house experiments were analyzed; these included a total of 16 females and 42 males, and were performed as described in this method section (see Figure 4C). The only difference with respect to methods is that three experiments were performed prior to transfection of the MRMT-1 cells line, and this study and one of the prior experiments were conducted after transfection of the MRMT-1 cells line. For all animals bodyweight and general health was monitored throughout the studies. All experiments were approved by the Danish Animal Experiments Inspectorate under the Danish Ministry of Food, Agriculture and Fisheries (2014-15-0201-00031), and were carried out in accordance to the guidelines of the Committee for Research and Ethical Issues of the International Association for the Study of Pain ^9^.

### Generation of MRMT1-Luc2

Generation of MRMT1 cells expressing reporter proteins was achieved by genomic integration of the Luc2-copGFP reporter gene construct using lentiviral transduction. The lentiviral vector contains the EF1α promoter sequence driving the equimolar expression of a codon-optimized firefly luciferase (Luc2) gene and a copepod green fluorescent protein (copGFP) thanks to the presence of a T2A sequence. Vector production and cell transduction were performed under appropriate biosafety level conditions (ML-II) in accordance with the National Biosafety Guidelines and Regulations for Research on Genetically Modified Organisms. Procedures and protocols were reviewed and approved by the LUMC Biosafety Committee (GMO permit 08-129). Vector production and transduction procedures have been described in detail elsewhere ^10^. In brief, cells were seeded in 24-well plate at a cell density of 7.5×10 ^4^ cells/well and maintained in a humidified incubator at 37°C and 5% CO _2_. After attachment was accomplished, the cells were transduced using a MOI (multiplicity of infection) of 1. Subsequently cells were FACS sorted for high expression of copGFP and cell culture was expanded for experiments.

### Cell culture

Syngeneic MRMT1-Luc2 rat mammary gland carcinoma cells (Leiden University medical Center, The Nederlands) were cultured in RPMI 1640 medium without phenol red (Invitrogen, Paisley, UK/Nærum, Denmark) supplemented with 10% heat-inactivated foetal bovine serum, 1% L-glutamine and 2% penicillin/streptomycin (Invitrogen, Paisley, UK/Nærum, Denmark). On the day of surgery, MRMT1-Luc2 carcinoma cells were released by brief exposure to 0.1% w/v trypsin-EDTA and centrifuged in medium for 3 min at 1200rpm. The pellet was washed twice with Hanks’ balanced salt solution (HBSS) without calcium, magnesium or phenol red (Invitrogen, Paisley, UK/Nærum, Denmark) and centrifuged for 3 min at 1200rpm. Cells were re-suspended in HBSS and kept on ice until use.

### Surgery

The inoculation of carcinoma cells was performed modified from 11 as previously described ^12^. Hence, following induction of isoflurane anaesthesia (induction 4%, maintenance 1.5–2%) the animal was placed with the abdominal side up. A small incision was made in a shaved and disinfected area on the anterior-medial surface of the tibia and the tibia carefully exposed. A hole was made in the tibia with a 0,7mm dental drill through which a thin polyethylene tube was fed 1cm into the proximal intramedullary cavity. Animals were injected with 5×10 ^3^ MRMT1-Luc2 carcinoma cells in 10µl HBSS. Following removal of the tube, the hole was plugged with bone restorative material (IRM, Dentsply, Surrey, UK/Vallensbæk, Denmark). The wound was irrigated with saline and closed with two metal clips. The animals were placed under a heat source until fully awake. Postoperative analgesia was administrated by injection of Rimadyl (s.c. 5mg/kg, Pfizer, Denmark) and application of 2% Lidocaine gel (AstraZeneca, Denmark) to the wound.

### Behavioral tests

Behavioral responses were assessed prior to surgery and on day 7, 10, 14, 17, 21 and 23. All animals were introduced to all behavioral tests 2–3 times prior to the start of the experiment. Animals reaching humane endpoint before day 23 (n=7) (predefined as limb use score 0) were euthanized by decapitation following brief exposure to isoflurane, and the behavioral scores from the last day carried forward for data analysis.

***Von Frey test.*** Mechanical hypersensitivity, detected by von Frey filaments (North Coast Medicinal, Inc., Camino Arroyo, Gilroy, CA, USA), was used as an indicator of early pain behavior. The threshold was determined by the up and down method, as described previously ^13^. Briefly, rats were placed in acrylic enclosures on a wire mesh floor and allowed to acclimatize for minimum 30 min. Starting at 6 g, filaments ranging from 0.4–26 g were applied to the plantar surface of the hind paw with a minimum of 3 min intervals between two stimulations. A stimulus was recorded as positive if paw withdrawal was observed within 3 s of stimulation with a given filament; if no response was observed it was recorded as negative. Following a positive response, the next stimulation was performed with a filament with a decreased bending strength, whereas a negative response was followed with stimulation with a filament with increased bending strength. According to the original protocol, optimal threshold calculation by this method requires 6 responses in the immediate vicinity of the 50% threshold, therefore recording of the 6 data points did not begin until the response threshold was first crossed (positive response changes to negative response or inverse). The 2 responses detecting the threshold were designated as the first 2 responses of the series of 6. In cases where continuous negative responses were observed to the maximum of the stimulus set, a value of 26 g was set as the cut-off and used as withdrawal threshold for data analysis. In all other cases the resulting pattern of positive and negative responses was calculated into a 50% response withdrawal threshold using the formula: 50% g threshold = (10Xf+kδ)/(10,000), where Xf = value (log unit) of the final von Frey hair used; k = tabular value for the pattern of positive/negative responses; and δ = mean difference (in log units) between filaments.

***Weight-bearing test.*** Rats were placed in the incapacitance tester (MJS Technology Ltd., Buntingford, Herfordshire, UK) and allowed to acclimatize until calm. Measurements were performed over a period of 4 s and in triplicates. An average weight-bearing ratio was subsequently calculated as the amount of weight placed on the cancer-bearing leg divided by the total amount of weight placed on both legs and used for data analysis.

***Limb use test.*** Rats were allowed to move freely in a transparent plastic cage without bedding (500 mm × 300 mm × 500 mm). Following 10 min of acclimation, each animal was observed for 3 min and assigned a limb use score from 3 to 0 as follows: 3: Normal use of leg, 2: mild or insignificant limping, 1: significant limping, 0: significant limping and part lack of use.

### Bioluminescence imaging

Animals were anesthetized in an induction chamber with 2.5% isoflurane (Isobar Vet; 100%, Nomeco, Copenhagen, DK). D-Luciferin (PerkinElmer, Skovlunde, Denmark) dissolved in PBS was administered by intraperitoneal injection (40mg/kg). 10 min after injection, animals were placed on their back in a nose cone in a Lumina XR instrument (Caliper Life Sciences, Teralfene, Belgium) and anesthesia was maintained with a 2.5% isoflurane/oxygen mix. Image capture was performed with binning: M(4), F/stop: 1 and exposure time from 1 s to 60 s according to the power signal. The signal was adjusted according to the exposure time prior to data analysis. For each animal, an average of three images was used for analysis. Between each capture, the animal was repositioned to minimize bias caused by placement of the animals in the machine. Bioluminescence images were analyzed using IVIS Imaging Software (Living Image©, version 4.0.0.9801; Caliper Life Sciences, Teralfene, Belgium). For each image, the threshold was adjusted to 35% of the signal, and the readout was measured in total flux, photos/s.

### X-ray analysis

X-Ray images were captured subsequent to the bioluminescence images. The severity of bone degradation was analyzed using ImageJ (ImageJ 1.47v). Each X-Ray image was calibrated to a standard aluminum wedge. The mean grayscale value of a standard region of interest within the trabecular bone of the proximal tibia was measured and the average of two corresponding background regions in the soft tissue proximate to tibia was subtracted. The grayscale value was translated into millimeter aluminum equivalents (mmAl) according to the standard wedge and used as estimate of the relative bone density of the distal femur. Data analysis was blinded for sex of the animals.

### Determination of active state

Animals with no bioluminescence signal or lack of osteolysis, hence no active cancer growth, were excluded from the active state group. All animals included in this study were assigned to an active or restored state based on presence of bioluminescence signal and osteolysis (active) or lack of bioluminescence signal and osteolysis (restored). Four previous in-house experiments were reanalyzed with regard to active and restored state based on presence bioluminescence signal and osteolysis or lack of bioluminescence signal and osteolysis, or based on osteolysis or lack of osteolysis alone. One study was similarly using bioluminescence as exclusion criteria, whereas the data from the three previous studies was analyzed with respect to lack of osteolysis. Lack of osteolysis was based on the last measure day, usually day 21–23, whereas one experiment was terminated at day 14, however on this day 10 out of 11 animals showed clear osteolysis.

### Adverse events

No adverse events were observed during the experiment.

## Statistical analysis

All analyses were blinded for the researchers. Analyses were performed using GraphPad Prism 6, (GraphPad Software, CA, USA), and for all data a 95% confidence interval was used as a measure of statistical significance. All data are expressed as mean ± standard error of mean (S.E.M.). *In vitro* analysis of bioluminescence signal was analyzed with linear regression. Behavioral, bone degradation and tumor progression data were analyzed using two-way repeated measure ANOVA followed by Bonferroni post-hoc test to compare baseline values to each additional time point, and in addition to compare females to males. Analysis of odds ratio of cancer clearance was tested using Chi-square test. Level of significance for all tests was set at */ ^#^p < 0.05, **/ ^##^p < 0.01, ***/ ^###^p < 0.001, ****/ ^####^p < 0.0001.

## Results

### Validation of bioluminescence emission in cancer cells

**Figure 1.**
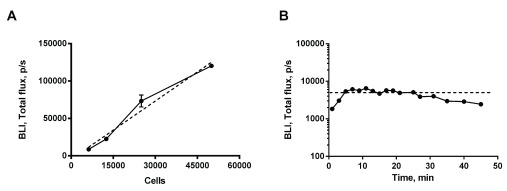
( **A**) *In vitro* quantification of bioluminescence signal. Linear regression demonstrated that the signal increased linearly with the number of cells (dotted line), p<0.0001. ( **B**) *In vivo* quantification. The bioluminescence signal increased during the initial 5min following i.p. injection. After 5min the signal reached a stable plateau lasting for approximately 20min. Results from two experiments.

The bioluminescence signal was evaluated to validate successful transfection of Luc2 into the MRMT-1 carcinoma cells. *In vitro*, the signal increased linearly with the number of cells, p <0.0001 ( Figure 1A). *In vivo*, i.p. administration of D-Luciferin induced a robust plateau of the bioluminescence signal 5 min after injection and lasting approximately 20min ( Figure 1B). Luc2 was hence successfully transfected into the carcinoma cells, linearly reflecting the number of living cells *in vitro*, and produced a robust signal *in vivo*.

### Sex-dependent variation in disease progression

**Figure 2.**
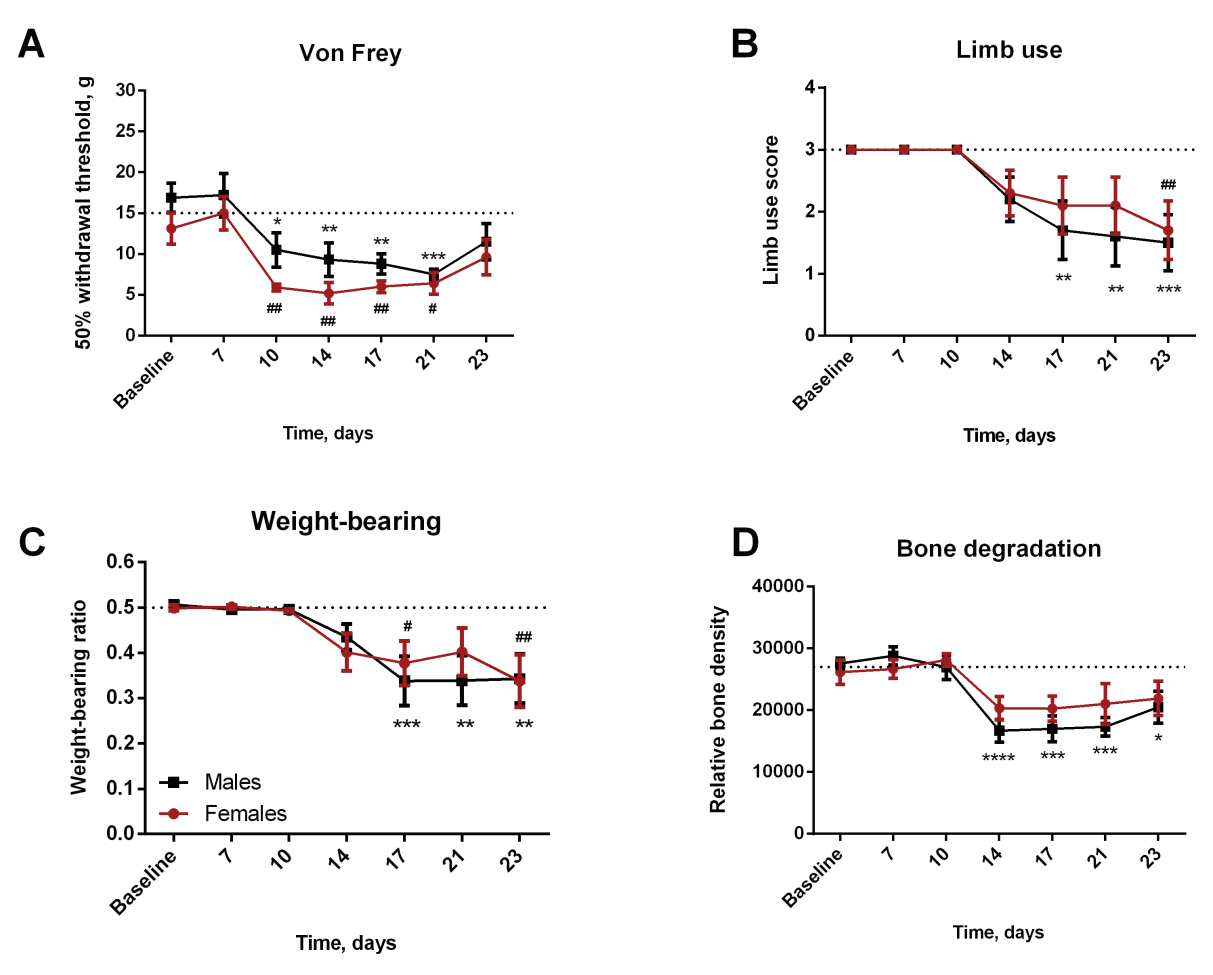
( **A**) A decrease in 50% withdrawal threshold was observed in both females and males as the disease progressed. ( **B** and **C**) Males demonstrated a significant decrease in limb use and weight-bearing ratio on day 17–23. In contrast, females showed significant decrease limb use only on day 23, and decreased weight-bearing on day 17 and 23. ( **D**) A significant decrease in relative bone density was observed in the males from day 14. A similar, yet insignificant, tendency was observed in the females. * ^/^** ^/^***=males compared to baseline, ^#/##^=females compared to baseline.

Pain behavior was quantified by detection of mechanical hypersensitivity, as well as limb use and weight-bearing deficits on days 7, 10, 14, 17, 21 and 23. Compared to baseline level, a significant decline in the 50% withdrawal threshold, reflecting mechanical hypersensitivity, was observed in both females and males from day 10 to 21 ( Figure 2A, Dataset 1: rawdata_vonfrey). Limb use scoring was significantly reduced only on day 23 for the females, but on day 17–23 for the males ( Figure 2B, Dataset 2: rawdata_limbuse). Decline in weight-bearing ratio was significantly reduced on day 17 and 23 for the females and on day 17–23 for the males ( Figure 2C, Dataset 3: rawdata_weight-bearing). No significant difference was observed between the sexes on any of the test days, however females tended to present with a less pronounced limb use and weight-bearing deficit in the later phase of disease progression, days 17–23 ( Figure 2B and C).

Rawdata_vonfrey Animals were placed in acrylic enclosures on a wire mesh floor and allowed to acclimatize for minimum 30 min. Starting at 6 g, filaments ranging from 0.4–26 g were applied to the plantar surface of the hind paw with a minimum of 3 min intervals between two stimulations. A stimulus was recorded as positive if paw withdrawal was observed within 3 s of stimulation with a given filament; if no response was observed it was recorded as negative. Following a positive response, the next stimulation was performed with a filament with a decreased bending strength, whereas a negative response was followed with stimulation with a filament with increased bending strength. The resulting pattern of positive and negative responses was calculated into a 50% response withdrawal threshold using the formula: 50% g threshold = (10Xf+kδ)/(10,000), where Xf = value (log unit) of the final von Frey hair used; k = tabular value for the pattern of positive/negative responses; and δ = mean difference (in log units) between filaments ^29^. Click here for additional data file. Copyright: © 2015 Falk S et al. 2015 Data associated with the article are available under the terms of the Creative Commons Zero "No rights reserved" data waiver (CC0 1.0 Public domain dedication).

Rawdata_limbuse Following 10 min of acclimation in a transparent plastic cage, each animal was observed for 3 min and assigned a limb use score from 3 to 0 as follows: 3: Normal use of leg, 2: mild or insignificant limping, 1: significant limping, 0: significant limping and part lack of use ^30^. Click here for additional data file. Copyright: © 2015 Falk S et al. 2015 Data associated with the article are available under the terms of the Creative Commons Zero "No rights reserved" data waiver (CC0 1.0 Public domain dedication).

Rawdata_weight-bearing http://dx.doi.org/10.5256/f1000research.6827.d96689
Animals were placed in the incapacitance tester and allowed to acclimatize until calm. Measurements were performed over a period of 4 s and in triplicates. An average weight-bearing ratio was subsequently calculated as the amount of weight placed on the cancer-bearing leg divided by the total amount of weight placed on both legs and used for data analysis ^31^. Click here for additional data file. Copyright: © 2015 Falk S et al. 2015 Data associated with the article are available under the terms of the Creative Commons Zero "No rights reserved" data waiver (CC0 1.0 Public domain dedication).

The relative bone density was significantly reduced compared to baseline measures in the males from day 14 and throughout the study ( Figure 2D, Dataset 4: rawdata_xray). A similar, however insignificant, tendency was observed in the females ( Figure 2D). No difference was found between males and females at any time point. Overall, the data suggest a less severe progression in females compared to males.

Rawdata_xray The severity of bone degradation was analysed. The x-ray was calibrated to a standard aluminium wedge. The mean grayscale value of a standard region of interest within the trabecular bone of the proximal tibia was measured and the average of two corresponding background regions in the soft tissue proximate to tibia was subtracted. The grayscale value was translated into millimeter aluminium equivalents (mmAl) according to the standard wedge and used as estimate of the relative bone density of the distal femur ^32^. Click here for additional data file. Copyright: © 2015 Falk S et al. 2015 Data associated with the article are available under the terms of the Creative Commons Zero "No rights reserved" data waiver (CC0 1.0 Public domain dedication).

### Females have increased capacity for recovery

**Figure 3.**
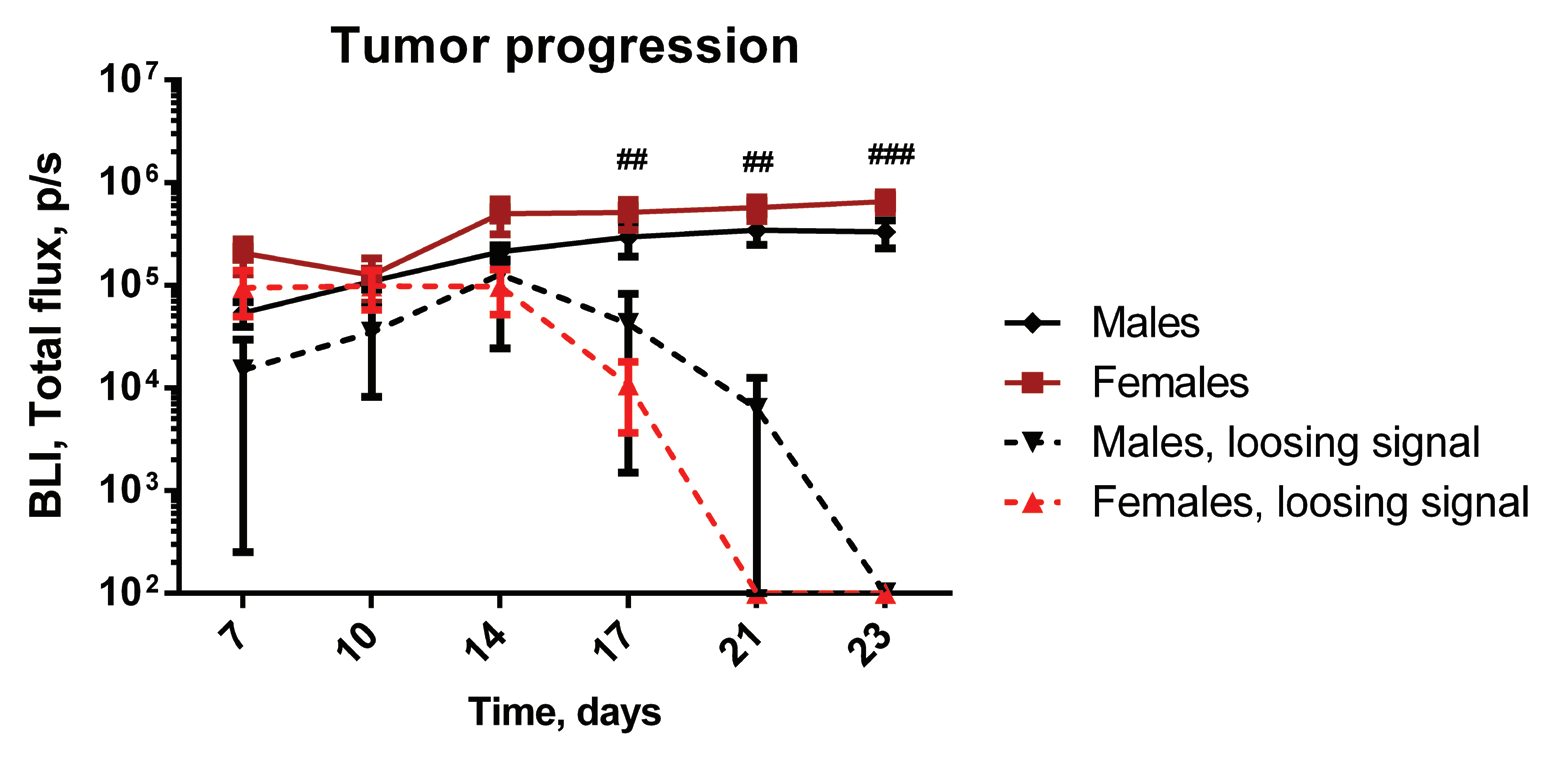
Bioluminescence (BLI) signal reflecting progression of tumor growth. The signal increases in both males and females during the initial 14 days, and reaches a plateau for the remaining of the study. From day 14 an increasing numbers of animals lost the bioluminescence signal. ^##/###^=females without signal compared to females with signal.

All animals had a detectable bioluminescent signal on days 7 and 10, hence demonstrating successful inoculation of living cancer cells ( Figure 3, Dataset 5: rawdata_BLI, Dataset 6: rawdata_bioluminescence). From day 14 an increasing number of animals lost the bioluminescence signal, suggesting recovery in a subset of animals. Compared to females with consistent bioluminescence signal, a subset of females losing bioluminescence signal displayed a significant decrease in the signal from day 17 ( Figure 3). A similar but later occurring tendency was observed in the males ( Figure 3).

**Figure 4.**
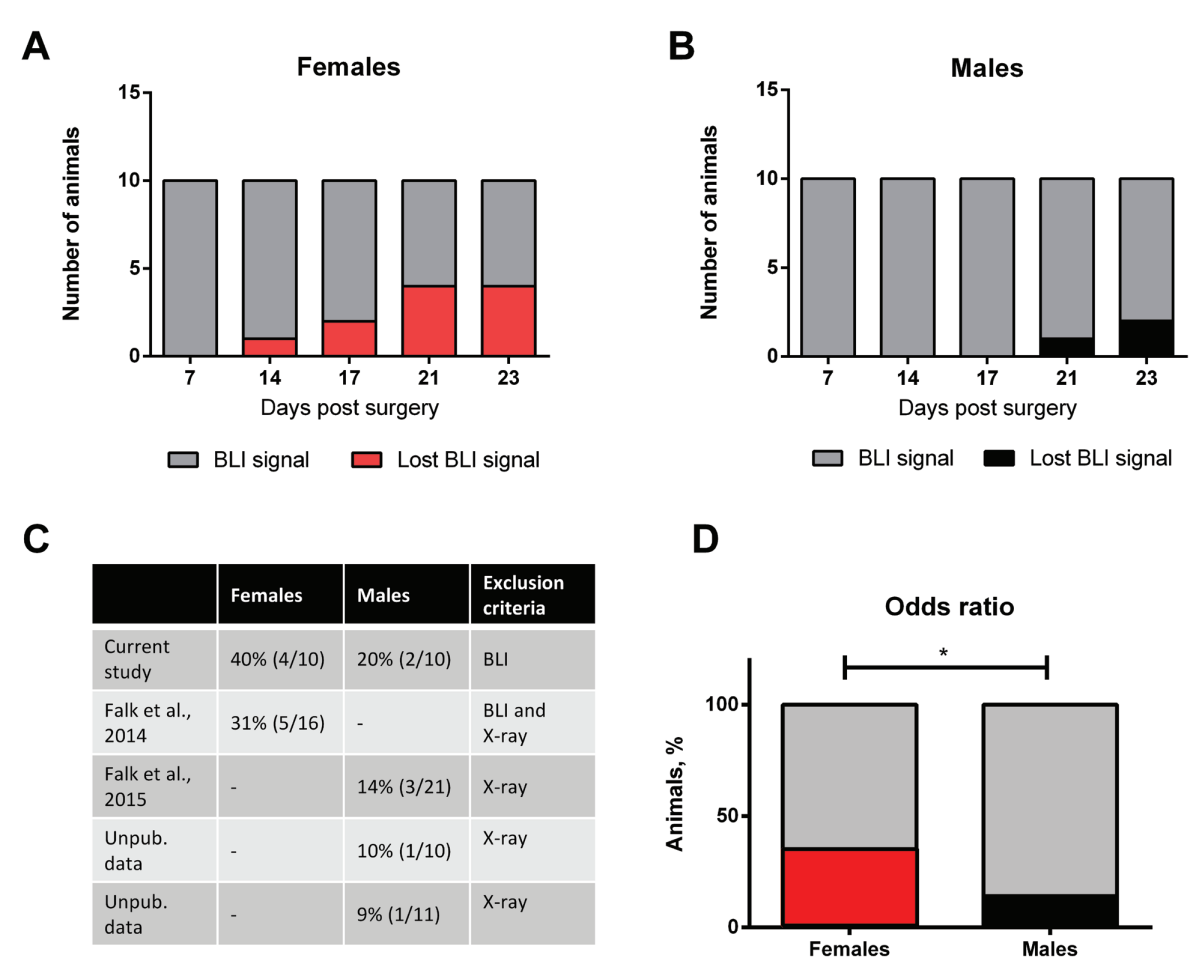
( **A** and **B**) Cumulative numbers of females and males losing bioluminescence signal over time. ( **C**) Numbers of animals with restored states in five independent in-house experiments. The recovery is based on either loss of bioluminescence signal or lack of osteolysis. ( **D**) A significant difference is found between the odds ratio of recovering in females and males. *=females compared to males.

**Supplementary figure S1.**
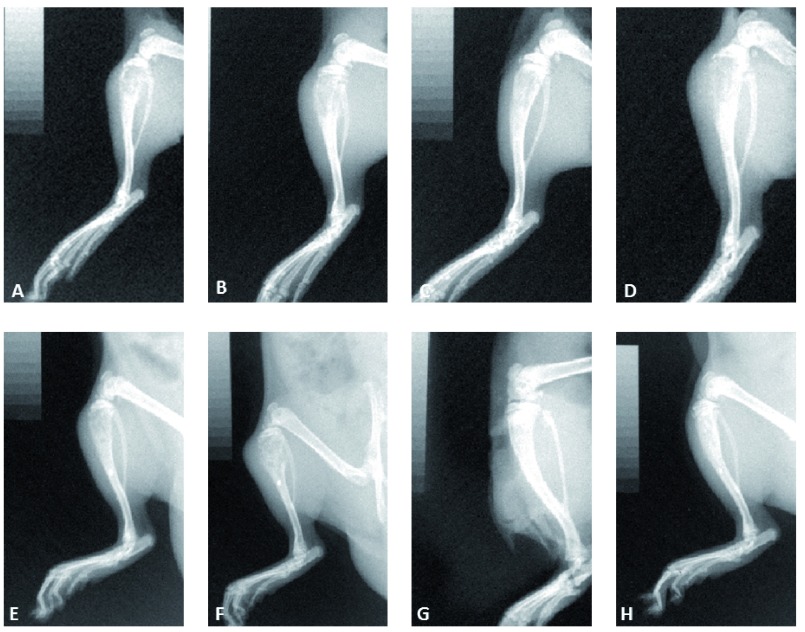
Representative images of animals demonstrating osteolysis ( **A**– **F**) and lack of osteolysis ( **G**, **H**).

**Supplementary figure S2.**
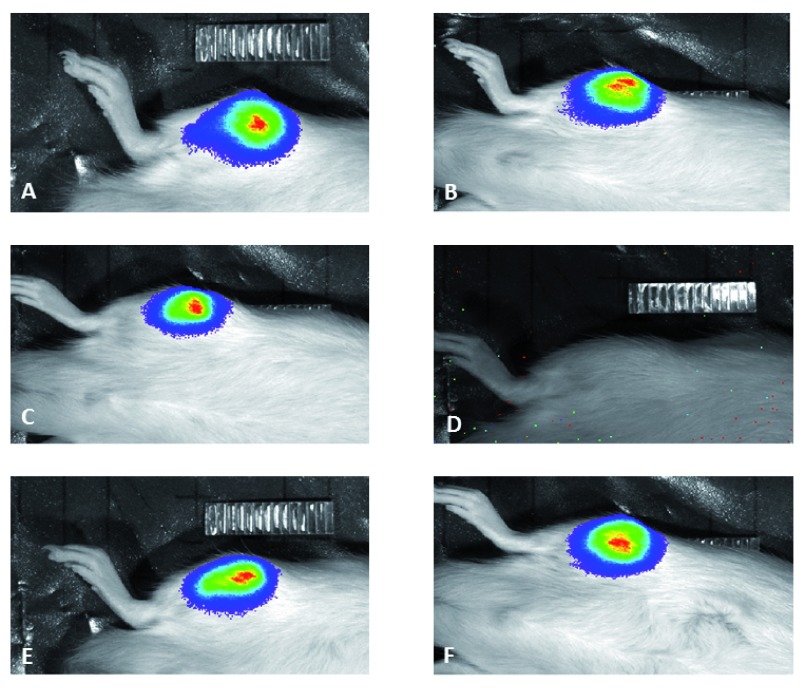
Representatives of animals demonstrating bioluminescence signal ( **A**– **C**, **E**, **F**) and lack of bioluminescence signal ( **D**).

Loss of bioluminescence signal was observed in 40% of the females, but only in 20% of the males ( Figure 4A and B). A significant trend for loss of signal was observed in both females and males, p=0.0079 and p=0.0353 respectively. A similar pattern in recovery was observed in previous independent in-house experiments. In these experiments, lack of osteolysis (determined by x-ray analysis) was used as an exclusion criterion. In one study, 5 out of 16 female animals displayed a lack of osteolysis ( Figure 4C) ^14^. Three additional experiments using male animals demonstrated lack of osteolysis at the end of the experiment in 3 out of 21, 1 out of 10 and 1 out of 11 animals, respectively ^12^ ( Supplementary figure S1 and Supplementary figure S2). Pooling the data from the five experiments demonstrated a significant difference in the odds ratio between females and males, reflecting a significantly greater proportion of females clearing active cancer growth compared to males, as indicated by loss of bioluminescence signal and/or lack of cancer-induced osteolysis, p=0.029, ( Figure 4D).

Rawdata_BLI Images were obtained 10min after i.p. injection of 40mg/kg Luciferase. Image capture was performed with binning: M(4), F/stop: 1 and exposure time from 1 s to 60 s according to the power signal. For each animal, an average of three images was used for analysis. For each image, the threshold was adjusted to 35% of the signal, and the readout was measured in total flux, photos/s ^33^. Click here for additional data file. Copyright: © 2015 Falk S et al. 2015 Data associated with the article are available under the terms of the Creative Commons Zero "No rights reserved" data waiver (CC0 1.0 Public domain dedication).

Rawdata_bioluminescence Representative bio-luminescence images ^34^. Click here for additional data file. Copyright: © 2015 Falk S et al. 2015 Data associated with the article are available under the terms of the Creative Commons Zero "No rights reserved" data waiver (CC0 1.0 Public domain dedication).

### Loss of bioluminescence signal is associated with reversal of disease progression

**Figure 5.**
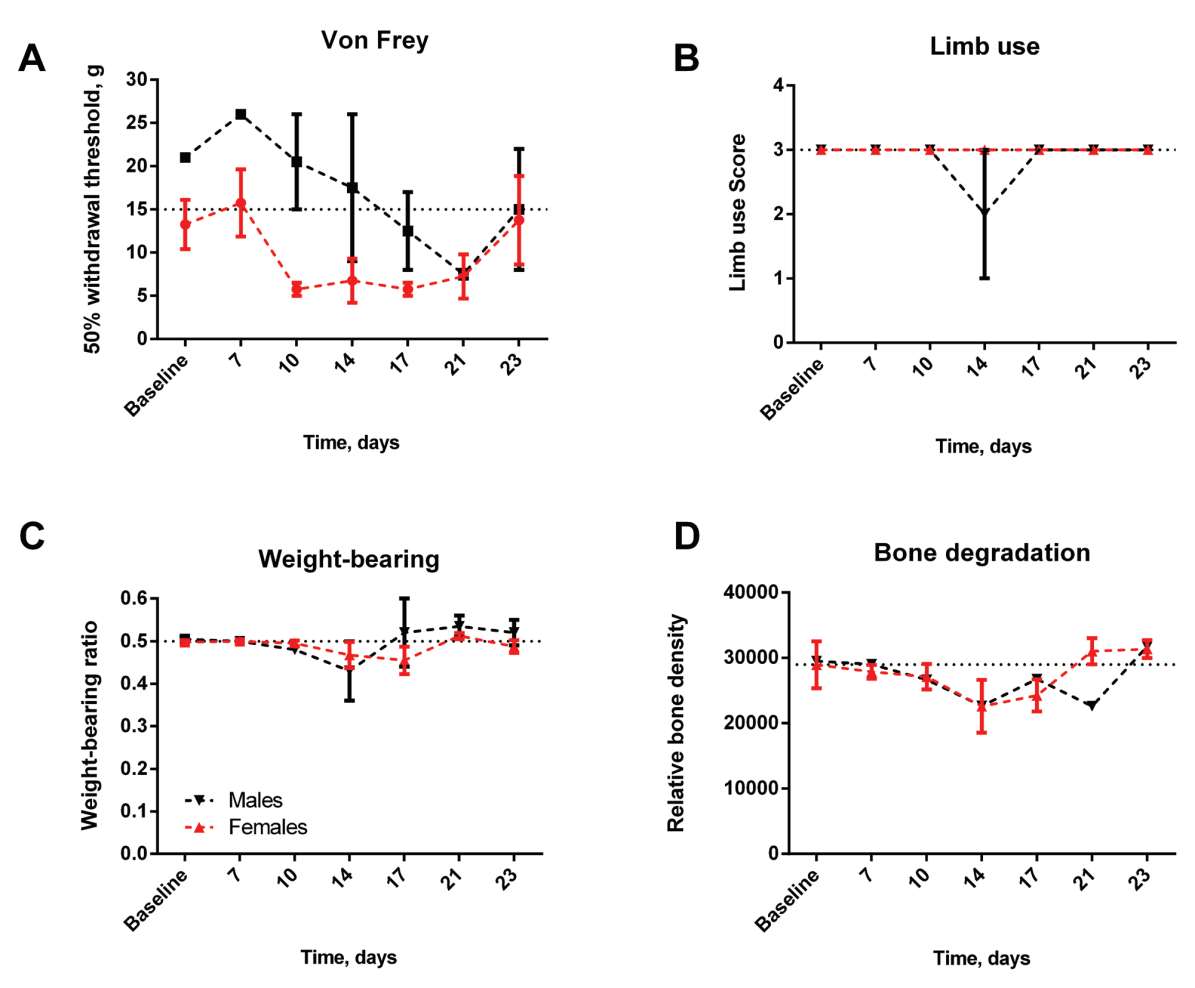
Pain-related behavior and relative bone density in animal losing bioluminescence signal during the experiment. ( **A**) A transient, yet insignificant, decrease was observed in the 50% threshold in females. ( **B**, **C**) Limb use and weight-bearing deficit was not observed in either females or males during the experiment. ( **D**) The relative bone density remained the same throughout the experiment in both males and females.

**Figure 6.**
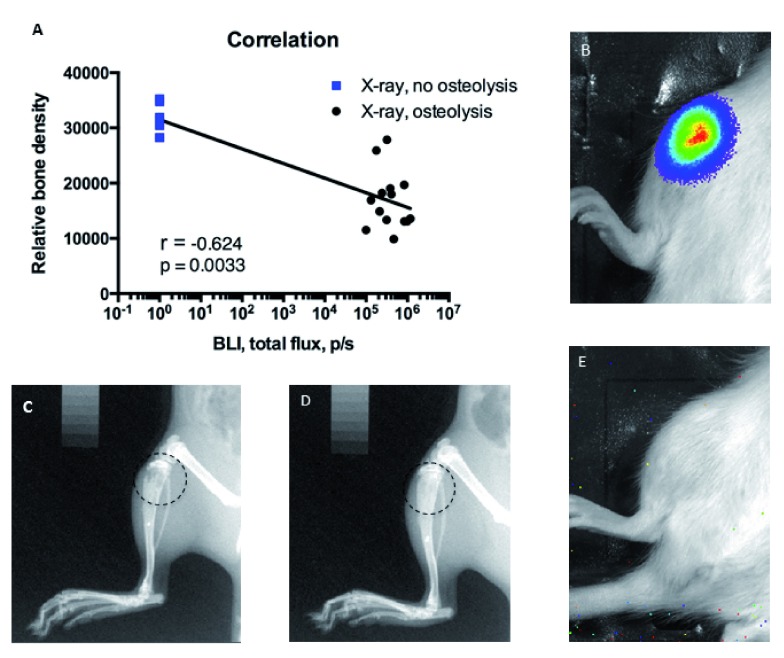
Correlation between relative bone density and bioluminescence signal. ( **A**) Animals without bioluminescence signal all had high relative bone density (blue), whereas animals with high bioluminescence signal demonstrated low relative bone density (black). Examples of osteolytic ( **C**) and non-osteolytic bone ( **D**). Examples of high bioluminescence signal ( **B**) and lack of signal ( **E**).

Despite the presence of living cancer cells during the initial 10 days, neither pain behavior nor relative bone density was significantly changed in animals losing bioluminescence signal ( Figure 5A–D). A transient but insignificant decrease in the 50% withdrawal threshold was observed in animals later losing bioluminescent signal ( Figure 5A, rawdata_vonfrey). In addition, a slight decrease in limb use, weight-bearing and relative bone density was observed on day 14, however the decrease was insignificant and returned to baseline levels on the next measured day ( Figure 5B–D, rawdata_limbuse, rawdata_weight-bearing, rawdata_xray). Overall this suggests initial normal disease progression, followed by subsequent recovery and hence normal relative bone density and lack of pain behavior. In addition, a strong correlation between bioluminescence signal and relative bone density was observed ( Figure 6A) supporting the division of animals into two groups; animals with a clear bioluminescence signal and osteolysis, indicating active cancer growth ( Figure 6A, B and C) and animals with no bioluminescence signal and no osteolysis, indicating recovery ( Figure 6A, E and D).

### Sex-difference screws progression of pain behavior

**Figure 7.**
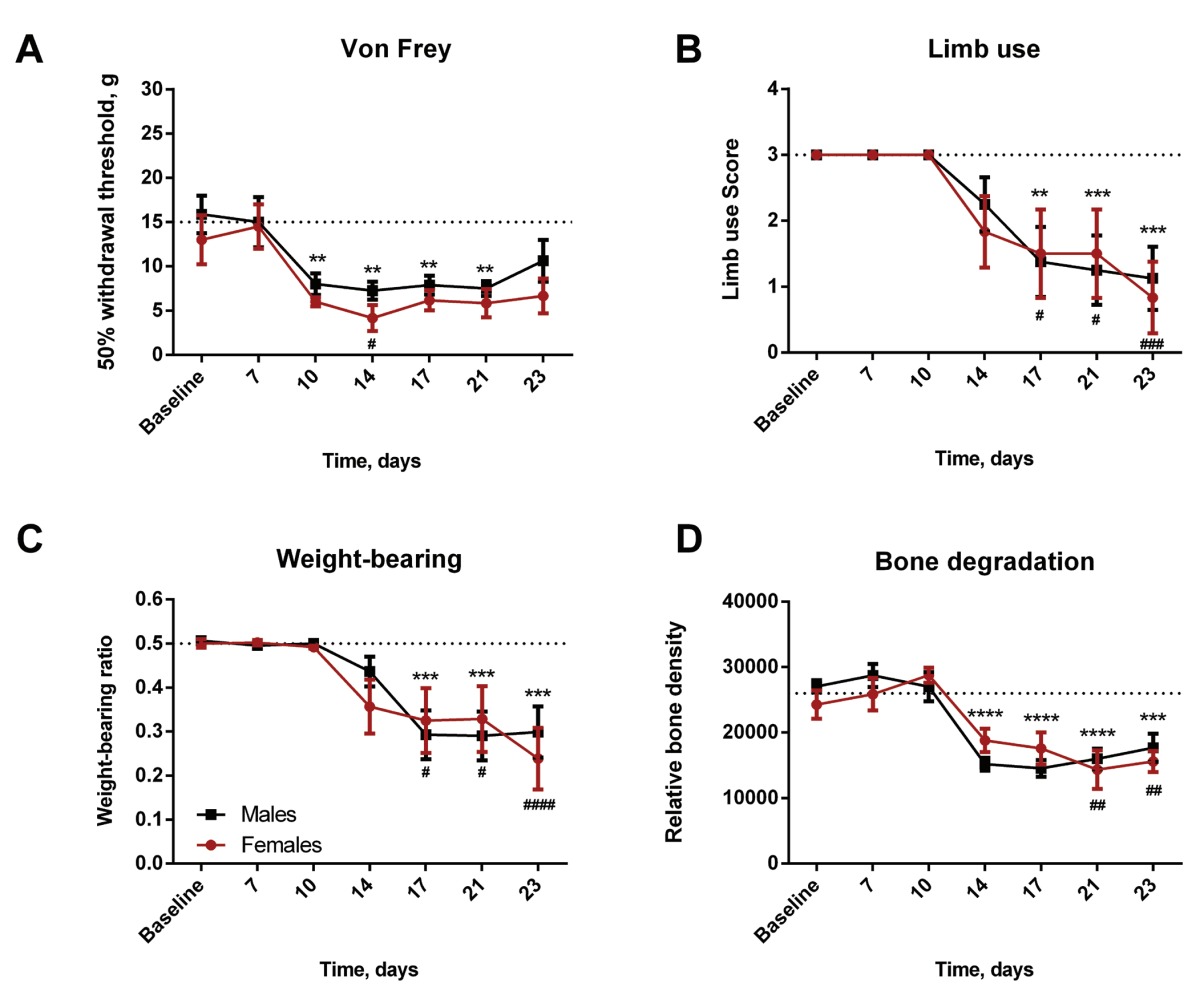
Pain-related behavior and relative bone density in animals with a consistent bioluminescence signal throughout the experiment. ( **A**, **B** and **C**) Both females and males demonstrate significant decreases in 50% withdrawal threshold, limb use score and weight-bearing ration. ( **D**) Both sexes show a similar extent of bone degradation. ** ^/^*** ^/^****=males compared to baseline, ^#/##/####^=females compared to baseline.

Exclusion of animals successfully recovering aligned the onset and severity of pain behavior in both sexes ( Figure 7A–C). In addition, a similar effect was seen on the relative bone density, reflecting similar bone degradation in both females and males with active growing cancer ( Figure 5D). No significant difference was observed between females and males at any time point.

## Discussion

To increase the translational potential of preclinical research, it is essential to continually focus on refining and optimizing the animal models used. Increased focus has been given to sex as a variable, initially driven by the contradictive issue that preclinical research has been biased towards use of male animals despite the fact the chronic pain is highly overrepresented in women ^8^. Although females are in most cases likely the most intuitive sex for models of metastatic breast cancer, such as the MRMT-1 model of cancer-induced bone pain, our data suggest that care should be taken when interpreting the data. Our data demonstrates that the females have an increased capacity for recovering from the disease state, reflected by loss of bioluminescence signal accompanied by normal limb use and weight-bearing ratio as well as normal relative bone density throughout the study.

The increased odds ratio for recovery in the females introduces a potential bias in interpretation of the data. Importantly, although there is no significant difference between females and male, the increased recovery in the females pulls the mean of the females’ readouts towards baseline to a much greater degree than the males, thereby increasing the risk of underestimating the severity of the disease in the females. In order to get a reliable model truly reflecting the human disease, it is critical to exclude the animals capable of recovery, as these animals are not reflecting the pain state observed in patients. This means that it is highly important to include a parameter for discrimination between an “active disease state” and a “recovered from disease state”. Inclusion of animals with recovered states clearly mask the severity of the disease in the females, as limb use is only significantly decreased on the last day and the relative bone density is not significantly affected at any time point ( Figure 2A and D). However, excluding females with recovered disease state reveals that females are in fact severely affected at an earlier time point, demonstrated by an significant decrease in limb use already on day 17 and in addition significant decreased relative bone density from day 21 ( Figure 7B and D).

In addition, our data demonstrates that bioluminescence signal is a reliable measure for the active disease state. All animals with continuous bioluminescence signal subsequently developed pain-related behavior and decreased relative bone density. In contrast, animals losing bioluminescence signal during the experiment did not develop pain-related behavior and demonstrated normal relative bone density. This is in agreement with other studies demonstrating that bioluminescence signal is predictable measure for quantification of cancer cells *in vivo* ^15, 16^. To further validate the recovery state, histology could be made to confirm that loss of bioluminescence signal is actually caused by clearance of the cancer cells and not just loss of signal from the cancer cells. However, since the first model of cancer-induced bone pain was developed, bone degradation has been demonstrated to associate with tumor burden numerous times ^17– 21^. In this study, the loss of bioluminescence signal is accompanied by lack of bone lesions and in addition lack of pain behaviors making it highly unlikely that there is still active cancer growth in the animals.

Importantly, this study emphasizes the need to consider sex-dependent variation beyond simple disease readouts. The data demonstrate that there are not significant difference between females and males with respect to pain behavior and bone degradation. However, there are sex-differences in the model which needs to be carefully considered when modeling cancer-induced bone pain in females, as the increased recovery rate in the females can potentially mask the progression. The specific mechanisms underlying the sex-dependent difference in recovery are currently not known, and requires an extensive investigation of hormonal and cellular responses. One potential cause could be sex-dependent differences in the immune responses. It is now generally accepted that inherent properties and influence of sex hormones induce more potent immune and inflammatory reactions in females compared to males ^22, 23^. An intuitive cause could therefore be, that the females’ increased recovery rate is linked to an increased immune response to inoculation with the cancer cells possible modulated by sex hormones. It should be noted that the capacity for recovering was seen regardless of whether the cancer cells expressed luciferase or not, and is thus not due to an immune reaction to the luciferase enzyme. However, as the MRMT-1 are estrogen receptor-postive ^20^, the relationship might not be so simple. Estrogen generally cause increase growth in estrogen receptor-positive cells ^24^, suggesting that the female immune system would have to fight a more rapidly growing tumor, which would suggest a lower recovery rate. A full understand of the involvement of sex hormones on the progression of MRMT-1 requires extensive studies; however our data suggest a minor role in the overall progression of cancer-induced bone pain in this model. This is also supported by the similar progression in males and female rats despite the estrogen receptor-positives status of the cells.

In addition, recent work from Sorge *et al.* demonstrate that different cells mediates pain hypersensitivity in female and male mice ^25^. They found that although the males and females developed the same degree of mechanical allodynia following nerve injury, there was a fundamental difference in the cellular involvement. Whereas microglia activity was required for mechanical pain hypersensitivity in males, this was not the case in females, where the hypersensitivity was driven by adaptive immune cells, likely T lymphocytes. Our data suggest that a similar phenomenon could be involved in progression of MRMT-1 induced cancer pain. In agreement with Sorge *et al.*, we find a similar pain response in females and males, however since there is a significant difference in the recovery rate between the sexes, it might indicate the different systems are mediating the pain state in female and male rats.

Another factor potentially affecting the sex-dependent effect might be the local microenvironment in the bone. The microenvironment around the tumor might be different due to nonspecific sex-differences, hence potentially affecting the growth of the tumor cells following inoculation ^26– 28^. Interestingly, the observed difference is likely species or cell line dependent. We have previously reported that in a similar model of cancer-induced bone pain, based on 4T1-luc2 mammary cancer cell inoculation in femur of BALB/cJ mice, females have a significantly greater initial bioluminescence signal compared to males. The females had, in addition, an earlier onset of pain behavior, but a similar bone degradation rate ^5^. This suggests that in the mouse model, intrinsic sex-dependent variation favors more aggressive progression in females compared to males, whereas the rat model displays better odds ratio for recovery in the females.

## Data availability

The data referenced by this article are under copyright with the following copyright statement: Copyright: © 2015 Falk S et al.

Data associated with the article are available under the terms of the Creative Commons Zero "No rights reserved" data waiver (CC0 1.0 Public domain dedication). 

*F1000Research*: Dataset 1. Rawdata_vonfrey, 10.5256/f1000research.6827.d96687 ^29^

*F1000Research*: Dataset 2. Rawdata_limbuse, 10.5256/f1000research.6827.d96688 ^30^

*F1000Research*: Dataset 3. Rawdata_weight-bearing, 10.5256/f1000research.6827.d96689 ^31^

*F1000Research*: Dataset 4. Rawdata_xray, 10.5256/f1000research.6827.d96690 ^32^

*F1000Research*: Dataset 5. Rawdata_BLI, 10.5256/f1000research.6827.d96691 ^33^

*F1000Research*: Dataset 6. Rawdata_bioluminescence, 10.5256/f1000research.6827.d96692 ^34^
