# Supplementary figures and images for: Effect of sex in the MRMT-1 model of cancer-induced bone pain
Source: F1000Res. 2015 Nov 16;4:445. Originally published 2015 Jul 31. [Version 3] doi: 10.12688/f1000research.6827.3 (PMC4706065; doi:10.12688/f1000research.6827.3)

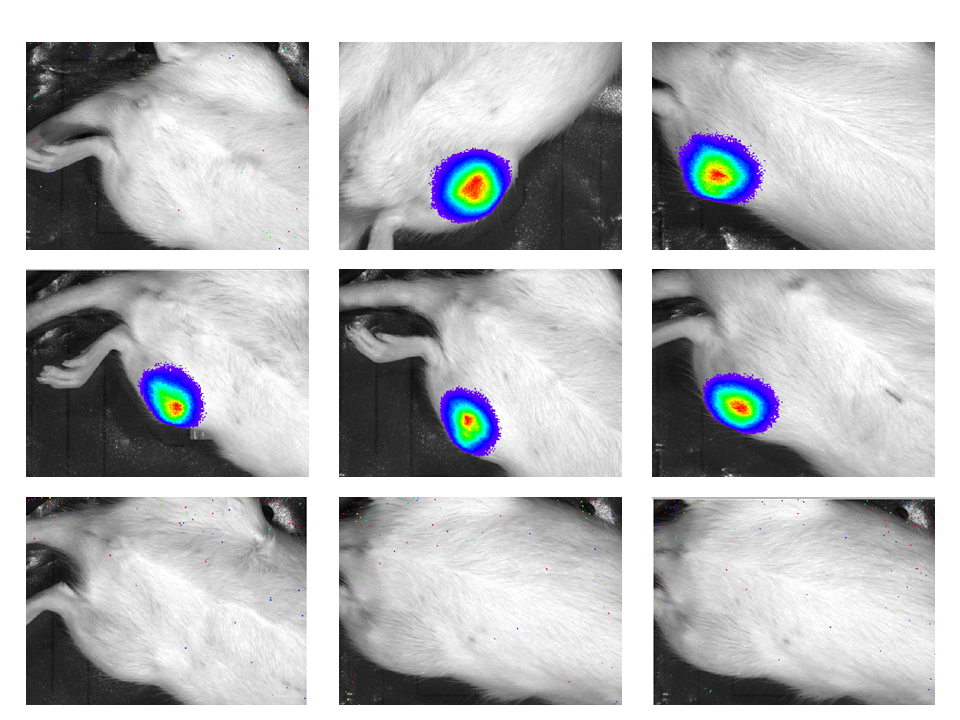

Supplement: Rawdata_bioluminescence — Representative bio-luminescence images 34. [file f1000research-4-7974-s0005.tgz › b067012d-dd48-462b-be06-01c28e06187e_Bioimages.tif]
