# Supplementary material for: Effect of sex in the MRMT-1 model of cancer-induced bone pain
Source: F1000Res. 2015 Nov 16;4:445. Originally published 2015 Jul 31. [Version 3] doi: 10.12688/f1000research.6827.3 (PMC4706065; doi:10.12688/f1000research.6827.3)
Supplement: Peer review history

# Peer review: Referee response for version 3

**DOI:** 10.5256/f1000research.7974.r11695

**Published:** 2015-12-22

**Authors:** Juan M Jiménez-Andrade

**Affiliations:** 1 Unidad Académica Multidisciplinaria Reynosa-Aztlán, Universidad Autónoma de Tamaulipa​s (UAT), Reynosa, Mexico

The authors have properly addressed all my previous concerns.

I have read this submission. I believe that I have an appropriate level of expertise to confirm that it is of an acceptable scientific standard.

# Peer review: Referee response for version 3

**DOI:** 10.5256/f1000research.7974.r11231

**Published:** 2015-11-30

**Authors:** Svetlana Komarova

**Affiliations:** 1 Faculty of Dentistry, McGill University, Montreal, QC, Canada

The revisions fully addressed my previous concerns.

I have read this submission. I believe that I have an appropriate level of expertise to confirm that it is of an acceptable scientific standard.

# Peer review: Referee response for version 2

**DOI:** 10.5256/f1000research.7621.r10387

**Published:** 2015-10-02

**Authors:** Svetlana Komarova

**Affiliations:** 1 Faculty of Dentistry, McGill University, Montreal, QC, Canada

I read the paper with interest and, although I do not always agree with the authors’ interpretation of the data, I found the study thought-provoking and analysis thorough and complex.

1. In my view, the main result of the study is that even though initial analysis demonstrates some difference between male and female animals inoculated with breast carcinoma cells, after the animals that spontaneously recovered from the disease were excluded, this difference between sexes was no longer evident. To me, this says that the process of cancer establishment in the bone is very similar in animals of different sex. This is a very interesting conclusion, however, to strengthen it I suggest to run the statistical analysis to directly compare male and female animals for figure 2 and 7, since it is possible that even though male and female animals behave (somewhat) differently compared to their respective baselines, there could be no significant difference between the sexes on Fig 2, which would dilute the impact of the conclusion. If that is the case, then the man focus of the study would become the difference in the percentage of animals spontaneously recovered from cancer inoculation.
2. I think the critical information missing from the description is the status of MRMT-1 cells with regard to estrogen and progesterone receptors. I have never worked with this cell line and I couldn’t find from the quick search if it is ER, PR positive or negative. I believe this information is critical both for interpretation of the difference in the cancer clearance rate between male and female animals, and the lack of difference in lesion progression between male and female animals.
3. I agree with Dr. Jiménez-Andrade that the sensitivity of the analysis of bone lesions based on grayness of X-rays is limited and that bone histomorphometry will significantly strengthen the conclusions.
4. I was confused with the last paragraph in Discussion on potential contributors to the difference in pain measures in male and female animals, when the conclusion of fig 7 is that there is no such difference.

I have read this submission. I believe that I have an appropriate level of expertise to confirm that it is of an acceptable scientific standard, however I have significant reservations, as outlined above.

# Response

**Published:** 2015-11-09

**Authors:** Sarah Falk

**Affiliations:** University of Copenhagen, Denmark

We thank Dr. Komarova for the helpful comments, and acknowledge that the study is somewhat untraditional in the sense that the main focus is on the increased variation observed in the females, which can potentially bias the model, rather than on a direct comparison on differences in readouts between males and females. Based on the reviewers useful criticism the following has been address and clarified in the discussion:

1. The reviewer is completely right; although the lack of difference between progression in female and male rats are on its own highly important for working with the model, the main result is not a direct comparison between sexes, but rather the differences in percentages of animals spontaneously recovering, and hence the indirect effect on variation in females and males. A statistical analysis comparing females to males was performed, and is mentioned in result section describing figure 2. The same analysis was performed for the data in figure 7, but was mistakenly not directly mentioned in the result section, this has been added. The main point is, as pointed out by the reviewer, the difference in ratio of spontaneous recovery between the sexes, which needs to be considered when working with the model in females, as the increased recovery rate can potentially mask the progression in females.  Elaboration on this has been added to the discussion.
2. The MRMT1 cells line has been reported to be estrogen positive[ref-1]. This seems counterintuitive as a negative status for the MRMT1 cells could possible explain the lack of difference in progression between the sexes, but here other factors seems more important. In addition, as estrogen is known to increase the growth of ER-positive tumors, the ER-positive status does not explain the increased recovery in females. If anything the ER-positive status of MRMT-1 cells should in theory have an opposite effect, hence decrease the likelihood of recovery in females. A section on this has been added to the discussion.
3. It is true that X-ray analysis for quantification of bone lesions provides much less detailed information as compared to histomorphometry. However X-ray analysis for quantification of bone lesions is widely used in the literature, and has been extensively demonstrated to associate with degree of tumor burden quantified by histology since the first model was described by Schwei in 1999 [ref-2] ^-^[ref-6]. Also, in this study the conclusion that the rats recover from the cancer is not solely based on the x-ray data but also on bioluminescene and pain-related behavior. A further discussion of this has been added to the discussion.
4. The paragraph regarding the recent study by Sorge et al is included to make attention to the fact that although the outcome of a condition may be the same, the underlying mechanisms can be different. Sorge et al demonstrate that although male and females displays the same degree of mechanical allodynia following nerve injury, the underlying mechanisms are different. We suggest that a similar phenomenon could be responsible for the data reported in this study.  Despite a similar outcome in terms of pain behavior the underlying mechanisms driving the pain might be mediated by different systems, which are likely linked to the observed sex-differences in recovery rates. This has now been clarified in the discussion.     

 [References]

[[1|authors=Doré-Savard/L;Otis/V;Belleville/K;Lemire/M;Archambault/M;Tremblay/L;Beaudoin/JF;Beaudet/N;Lecomte/R;Lepage/M;Gendron/L;Sarret/P|title=Behavioral, medical imaging and histopathological features of a new rat model of bone cancer pain|source=PLoS One|year=2010|vol=5|issue=10|pmid=21048940|pmcid=2966439|doi=10.1371/journal.pone.0013774]]

[[2|authors=Schwei/MJ;Honore/P;Rogers/SD;Salak-Johnson/JL;Finke/MP;Ramnaraine/ML;Clohisy/DR;Mantyh/PW|title=Neurochemical and cellular reorganization of the spinal cord in a murine model of bone cancer pain|source=J Neurosci|year=1999|vol=19|issue=24|fpage=10886|lpage=10897|pmid=10594070]]

[[3|authors=Luger/NM;Mach/DB;Sevcik/MA;Mantyh/PW|title=Bone cancer pain: from model to mechanism to therapy|source=J Pain Symptom Manage|year=2005|vol=29|issue=5 Suppl|fpage=S32|lpage=S46|pmid=15907645|doi=10.1016/j.jpainsymman.2005.01.008]]

[[4|authors=Hu/Z;Gerseny/H;Zhang/Z;Chen/YJ;Berg/A;Zhang/Z;Stock/S;Seth/P|title=Oncolytic adenovirus expressing soluble TGFβ receptor II-Fc-mediated inhibition of established bonemetastases: a safe and effective systemic therapeutic approach for breast cancer|source=Mol Ther|year=2011|vol=19|issue=9|fpage=1609|lpage=1618|doi=10.1038/mt.2011.114|pmid=21712815|pmcid=3182349]]

[[5|authors=Feng/S;Zhu/G;McConnell/M;Deng/L;Zhao/Q;Wu/M;Zhao/Q;Wang/J;Qi/J;Li/YP;Chen/W|title=Silencing of atp6v1c1 prevents breast cancer growth and bone metastasis|source=Int J Biol Sci|year=2013|vol=9|issue=8|doi=10.7150/ijbs.6030|pmid=24155661|pmcid=3805834]]

[[6|authors=Juárez/P;Mohammad/KS;Yin/JJ;Fournier/PG;McKenna/RC;Davis/HW;Peng/XH;Niewolna/M;Javelaud/D;Chirgwin/JM;Mauviel/A;Guise/TA|source=Cancer Res|year=2012|vol=72|issue=23|fpage=6247|lpage=6256|doi=10.1158/0008-5472.CAN-12-1444|pmid=23002206|pmcid=4447239]]

# Response

**Published:** 2015-11-27

**Authors:** Komarova Svetlana

**Affiliations:** McGill University, Canada

Thank you for your response. I believe that new discussion provides a significant and novel focus for the study.

# Peer review: Referee response for version 1

**DOI:** 10.5256/f1000research.7341.r10035

**Published:** 2015-08-20

**Authors:** Juan M Jiménez-Andrade

**Affiliations:** 1 Unidad Académica Multidisciplinaria Reynosa-Aztlán, Universidad Autónoma de Tamaulipa​s (UAT), Reynosa, Mexico

This is very interesting and well presented study; however the data does not support the title and conclusions at all of the present study. For this reviewer it is unclear what is the functional implication of females having an increased capacity for recovering from cancer state, given that there are non-significant differences in terms of pain behaviors and bone degradation between females and male rats. I think the authors are overstating the results and the results are not properly interpreted.

Additionally, the authors have not considered that the loss of bioluminescence signal over time may due to gender-related differences in terms of bioavailability and excretion of D-Luciferin. In fact, the authors are strongly recommended to present the *in vivo* quantification of bioluiminescence signal after i.p. injection (as presented in figure 1B) in both females and males.

Finally, a histopathological analysis of the bone is strongly needed in order to suggest that females rats are recovering faster than male rats. 

**EDIT 21/08/2015: This review was mistakenly published with a ‘Not Approved’ status, this has now been amended to an ‘Approved with Reservations’. The text has not been changed in any way.**

I have read this submission. I believe that I have an appropriate level of expertise to confirm that it is of an acceptable scientific standard, however I have significant reservations, as outlined above.

# Response

**Published:** 2015-09-10

**Authors:** Sarah Falk

**Affiliations:** University of Copenhagen, Denmark

Dear Professor Jiménez-Andrade

Thank you for your very constructive review. We have taken your comments into carefully consideration, and made the following changes:

We have changed the title to “Effect of sex in the MRMT-1 model of cancer-induced bone pain”, as we realize that the original title could be misleading.

Regarding the functional implication of the study, we have extended the discussion to clarify that the main result of the study is the increased capacity for recovery from the tumor-induced disease in the female rats compared to the male rats. This can potentially affect the interpretation of data produced with the model.

The last line in the abstract is in addition changed to “interpretation of data”, to emphasize that the increased capacity for recovery do not affect the overall progression in cancer-bearing animals, but that the recovery in some animals can mask the actual effect of the cancer, as the data from the recovered animals will shift the mean towards baseline. 

Sex-related differences in terms of bioavailability and excretion of D-Luciferin should not affect the result in the study. The bioluminescence emission is based on acute injection of D-Luciferin at a level that saturates the system. Bioluminescence signal is measure 10min past injection on each measure day, meaning that if there were sex-differences in bioavailability and excretion is should be seen on all measuring day, and not only in a subset of animals from day 17 and beyond. Also as can be seen in supplement figure S2 the loss of signal is seen as an all-or-non response; either there is a signal or no signal at all. If the loss of bioluminescence signal were caused by changes in bioavailability and excretion in a subset of animals a more gradient decrease in signal would be expected and not a complete loss of signal as seen in these animals.

Additional histopathological analysis could be performed, however since loss of bioluminescence signal is accompanied by both lack of pain behavior and osteolysis it is unlikely that the animals should have living cancer cells in tibia. To emphasis this point, we have added a section in the discussion.
